# Threshold-Based BRISQUE-Assisted Deep Learning for Enhancing Crack Detection in Concrete Structures

**DOI:** 10.3390/jimaging9100218

**Published:** 2023-10-10

**Authors:** Sanjeetha Pennada, Marcus Perry, Jack McAlorum, Hamish Dow, Gordon Dobie

**Affiliations:** 1 Department of Civil and Environmental Engineering, University of Strathclyde, 75 Montrose St., Glasgow G1 1XJ, UK; 2 Department of Electronic & Electrical Engineering, University of Strathclyde, 204 George St., Glasgow G1 1XW, UK

**Keywords:** image quality assessment, deep learning, VGG16, image processing, structural health monitoring, neural networks, binary classification, data cleaning, BRISQUE, concrete crack detection

## Abstract

Automated visual inspection has made significant advancements in the detection of cracks on the surfaces of concrete structures. However, low-quality images significantly affect the classification performance of convolutional neural networks (CNNs). Therefore, it is essential to evaluate the suitability of image datasets used in deep learning models, like Visual Geometry Group 16 (VGG16), for accurate crack detection. This study explores the sensitivity of the BRISQUE method to different types of image degradations, such as Gaussian noise and Gaussian blur. By evaluating the performance of the VGG16 model on these degraded datasets with varying levels of noise and blur, a correlation is established between image degradation and BRISQUE scores. The results demonstrate that images with lower BRISQUE scores achieve higher accuracy, F1 score, and Matthew’s correlation coefficient (MCC) in crack classification. The study proposes the implementation of a BRISQUE score threshold (B_*T*)_ to optimise training and testing times, leading to reduced computational costs. These findings have significant implications for enhancing accuracy and reliability in automated visual inspection systems for crack detection and structural health monitoring (SHM).

## 1. Introduction

Monitoring the health of civil engineering infrastructure is crucial to ensure safety and longevity. One of the most important aspects of Structural Health Monitoring (SHM) is the ability to detect, locate, and monitor damage [1,2,3]. The one major damage affecting civil infrastructure safety is a crack. Regular inspection and maintenance of civil infrastructures, such as bridges, pavements, and underground structures, are essential to ensure optimal functioning [4,5,6]. Traditional inspection methods by skilled inspectors have limitations, prompting the need for automated inspections using robotics. This approach offers several benefits, including reduced costs, minimal disruptions, and lower risk [7,8,9,10].

Automatic defect detection can be separated into two main approaches: white-box and black-box techniques [11]. The former uses algorithms such as edge detectors and thresholding [12], whereas the latter employs machine learning and artificial neural networks [13]. Both methods have unique benefits that depend on the application. Generally, the black box is more effective for initial detection, while the white box is better suited for pixel-by-pixel segmentation [14].

The VGG deep convolutional neural network architecture, developed by the Visual Geometry Group (VGG), has a well-known success rate in identifying and categorizing cracks in concrete structures [15]. By incorporating transfer learning and fine-tuning to the VGG architecture, we significantly enhanced detection accuracy, thereby making a valuable contribution to the field of structural health monitoring and image quality assessment.

An adaptive lighting platform integrated with robotics is utilised to capture images of concrete samples in low-light environments to facilitate crack detection. This system features a machine-vision camera fitted with four adjustable arms, each fitted with LED strips [16]. The images are captured using diffuse lighting. However, using robotics and cameras in low-light environments can introduce potential challenges, such as blur and noise during image capture. These factors can significantly affect the quality of the captured images and affect the model’s recognition capability during the training and testing phases [17]. So, it is essential to evaluate the quality of these images before feeding them to the image processing algorithms, which can be achieved using a no-reference image quality assessment (NR-IQA) technique called the Blind/Referenceless Image Spatial Quality Evaluator (BRSIQUE) [18].

**Figure 1:**
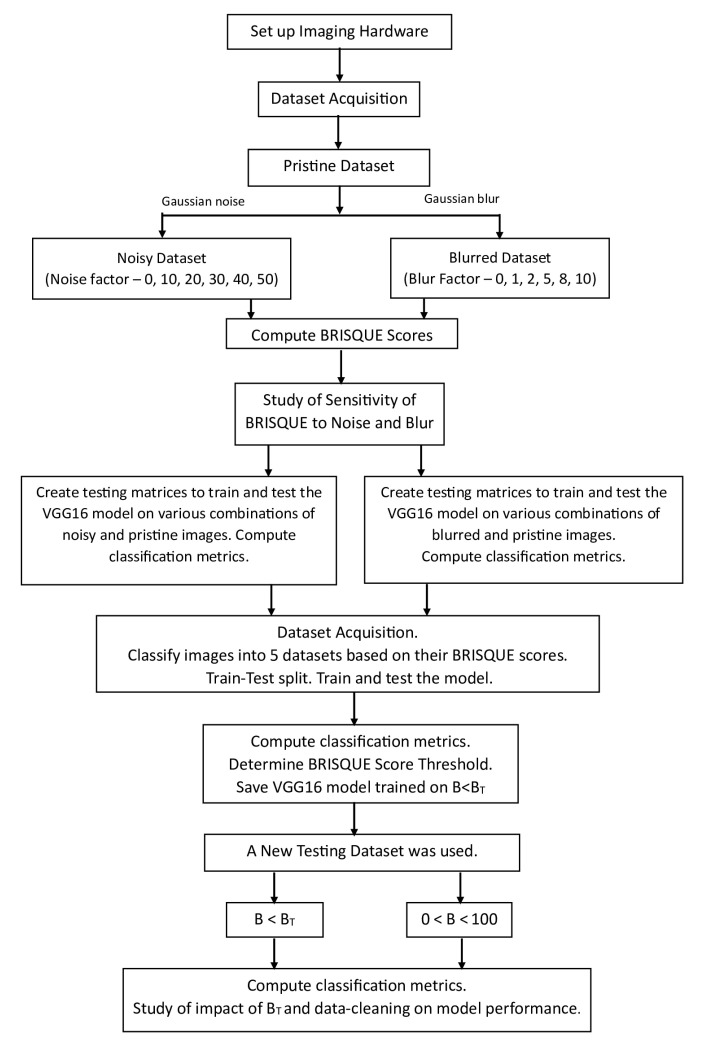
Workflow overview of threshold-based BRISQUE-assisted deep learning for enhancing crack detection in concrete structures.

The aim of this study is two-fold. Firstly, it examines the correlation between the BRISQUE image quality metric and the performance of the CNN model in crack detection. The model’s performance will be assessed on pristine images and those with artificially introduced Gaussian noise and Gaussian blur images. The aim is to better understand how image degradation affects crack classification accuracy, F1 score, and Matthew’s correlation coefficient (MCC). Secondly, it proposes a methodology to determine a BRISQUE score threshold (B${}_{T}$) to identify and discard low-quality images from the datasets. Also, the impact of B${}_{T}$ and data cleaning on the performance of the model is studied by evaluating the trained threshold-based BRISQUE-assisted deep learning model on an independent testing set. The workflow of the entire process is clearly shown step-by-step in Figure 1.

## 2. Background

For major civil and construction assets, semi-autonomous or remote inspections are sometimes carried out with Unmanned Aerial Vehicles (UAVs) or wholesale robotic platforms fit with inspection equipment, as reviewed in [19]. State-of-the-art research showcases the ability to capture cracked surfaces using moving vehicles [20] or with UAVs [21]. In the majority of the reported literature, the process of capturing images is typically conducted separately from the crack detection phase. In an industrial setting, this would mean that any errors or inaccuracies occurring during image capture could not be corrected or adjusted. This would cause either imperfect analysis (requiring error correction) or overlooking significant defects altogether. An innovative system has been employed in our study to capture images of concrete samples in low-light environments, making it easier to identify cracks.

**Figure 2:**
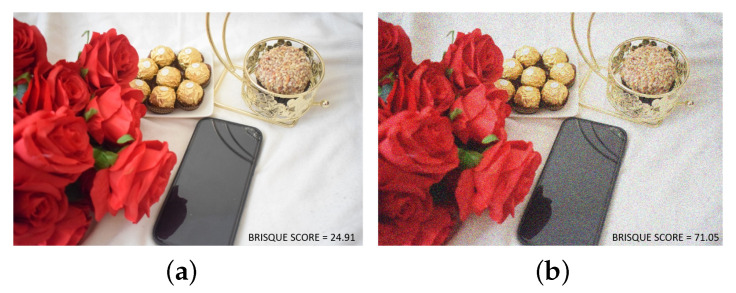
Comparison of image quality using BRISQUE scores. (**a**) Natural image with a BRISQUE score of 24.91, indicating better image quality. (**b**) Noisy image with a BRISQUE score of 71.05, indicating poor image quality.

Several factors affect the quality of an image when captured using a camera, including lighting conditions, camera settings, motion blur, lens distortion, and noise. A degraded image refers to a modified version of the original (pristine) image that has been affected by various factors such as blur and noise, either by natural factors or deliberately in post-processing. Since it is uncertain whether a captured image will be natural, noisy, blurry, or distorted in any other way, assigning a score based on the image quality can help in determining the suitability of the image for training or testing the CNN models. Figure 2 provides an example of a raw image (natural image) and a degraded image (noisy image), along with their BRISQUE scores. Therefore, accurate image quality measurement is essential for developing and evaluating algorithms in various fields. The goal of IQA is to provide methods for measuring image quality in a way that aligns with human perception [22,23].

### 2.1. Image Quality Assessment (IQA)

Image quality assessment is the process of evaluating the quality of an image. It is widely used in machine learning, computer vision, neural physiology, image processing, and other domains where image quality plays an important role [24]. There are two types of IQA methods.

#### 2.1.1. Subjective Methods

Subjective image quality assessment methods involve gathering individual opinions on the quality of images using a five-level rating scale without participants having full access to the scores or information from others. By averaging the scores provided by the participants, a reference point for image quality is established, and collecting opinions from a larger number of individuals enhances the reliability of these results [25].

In practical applications, subjective evaluations are often considered the most accurate for assessing image quality. However, they are expensive, time-consuming, and influenced by various factors. To address these issues, objective IQA methods have been developed, which use numerical methods and mathematical models to predict the perceptual quality of the images [26].

#### 2.1.2. Objective Methods

The development of accurate mathematical models to predict the quality of images similar to human observers has become crucial in objective IQA methods. These models aim to replicate human quality predictions and provide efficient and cost-effective image quality evaluation [27]. Objective IQA methods extract features from the image and analyse them using a quality score [28]. IQA research is typically classified into three frameworks: Full-Reference (FR), Reduced-Reference (RR), and No-Reference (NR) or Blind IQA [29]. FR methods require a reference image, while RR methods use extracted features from the reference image to evaluate image quality. On the other hand, NR methods can assess image quality without the need for a reference image. All three approaches have their advantages, but obtaining the original reference image in real-world applications can be challenging. In such scenarios, using a no-reference metric is more efficient and practical because it does not rely on the availability of the reference image [30,31,32].

### 2.2. BRISQUE Method

One way to assess the quality of an image is by using no-reference metrics, such as the Blind or No-Reference IQA technique called the Blind or Referenceless Image Spatial Quality Evaluator (BRISQUE). Numerous studies have assessed the effectiveness of BRISQUE techniques in evaluating the quality of medical images [27] and concrete crack images [33].

In Ref. [34], the live road footage captured by a camera mounted on a vehicle was evaluated using the BRISQUE algorithm to determine its quality for road damage detection and driver notification. The quality of raw IRT images and their potential distortions caused by preprocessing were evaluated in [35] using BRISQUE as a referenceless image quality assessment metric. Ref. [36] utilised BRISQUE as a no-reference image quality assessment (IQA) technique to evaluate the effectiveness of preprocessing in improving the quality and visibility of low-light images. The focus was on enhancing the visibility for identifying faulty porcelain insulators in a low-voltage power distribution system. Only a limited number of studies have examined the effectiveness of the BRISQUE technique in evaluating the quality of concrete crack images.

BRISQUE employs a support vector regression (SVR) model trained on images with known distortions, such as blur, noise, and artefacts, to compute the image quality score. It compares the distribution of the mean subtracted contrast normalisation (MSCN) of the subject image to the database images. The MSCN of a pixel at location (i, j) is defined by Equation (1), where I represents the intensity of the pixel, μ is the local mean, and σ is the local variance in intensity of the surrounding pixels.

(1) $$MSCN\left(i,j\right)=\frac{I(i,j)-\mu (i,j)}{\sigma (i,j)+1}$$

The process of identifying image distortions using the BRISQUE model involves generating a histogram of MSCN values. This model can detect various distortions such as blur, noise, and compression by analysing the shape and variance of the distribution. The model then compares these variables to an image database and assigns a score ranging from 1 to 100, with lower scores indicating better image quality. A score close to 0–30 suggests a good (high) image quality, while a score greater than 40 indicates a poor (low) image quality. An example of this is shown in Figure 2.

This study explores how different types of image degradations, such as Gaussian noise and Gaussian blur, affect the BRISQUE scores. The main goal is to use BRISQUE scores to assess the quality of original and artificially degraded images and determine the stability of neural network models. Additionally, the research seeks to implement a BRISQUE score thresholding technique to discard low-quality images and improve the performance of the image-based crack detection model.

### 2.3. Convolutional Neural Network (CNN)

CNNs are widely used in computer vision and image classification applications for detecting features and patterns within an image. The VGG architecture, a deep CNN model, has been proven effective in identifying and classifying cracks in concrete structures. The VGG16 model, which has 13 convolutional layers and three fully connected layers, is used in this study as a pre-trained model. This model was originally trained on the ImageNet dataset and can process images that are 224 × 224 pixels with three channels. Its training was based on the ImageNet dataset [37,38].

## 3. Methodology

The primary aim of this research is to assess the image quality by assigning a BRISQUE score to each image captured. Then, a VGG16 neural network model is utilised to train these images to detect cracks on concrete surfaces. This study investigates how well the deep learning models can detect cracks and analyse their relationship with BRISQUE scores. The study also highlights the benefits of using a BRISQUE score threshold (B${}_{T}$) to improve the model’s ability to recognise cracks effectively.

### 3.1. Overview

**Figure 3:**
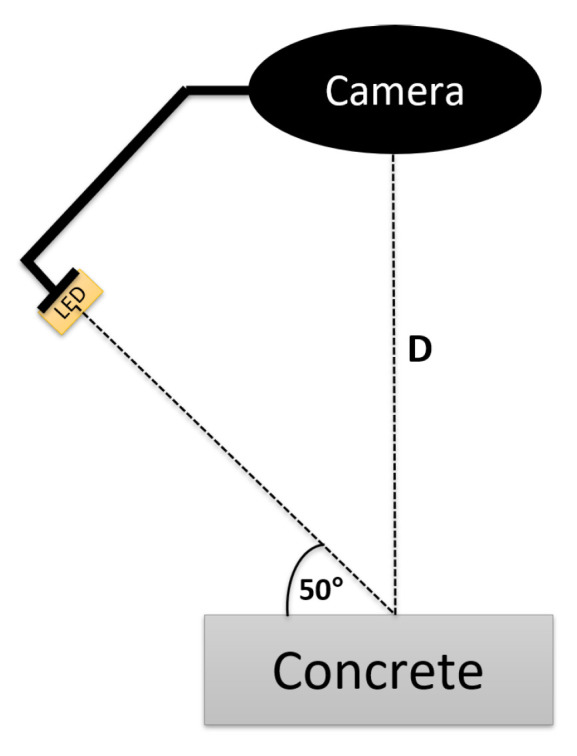
Specialized inspection equipment with single adjustable arm for 50-degree lighting projection onto concrete surface.

Our inspection device is built to capture images under diffused lighting conditions, utilizing a machine-vision camera surrounded by four adjustable arms, each fitted with RGB LED strips. These lighting arms are positioned at a 50 degree angle of incidence relative to the concrete surface. An illustration of this setup, using a single arm, is presented in Figure 3, where D represents working distance, which is explained in subsequent sections.

#### 3.1.1. Image Capture

Engineering standards typically state that crack widths of 0.1 mm to 0.3 mm in concrete structures should be identified for further action [39]. We have therefore chosen to work towards a minimum spatial resolution of ≤0.1 mm/pixel during image capture.

#### 3.1.2. Hardware and Settings

**Figure 4:**
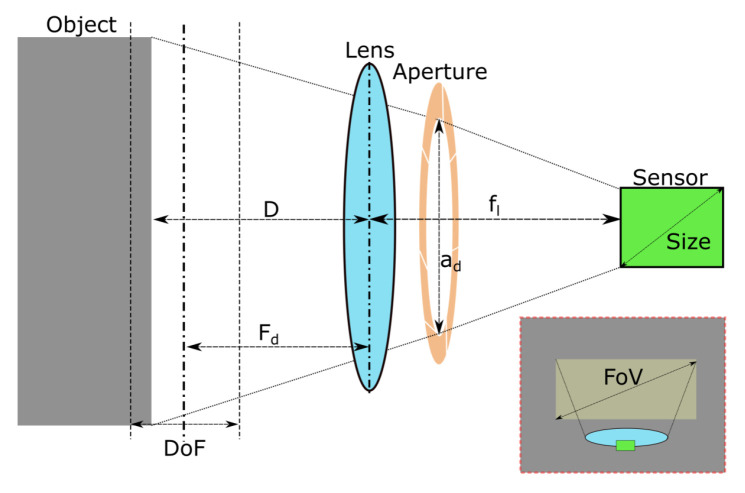
Illustration of variables during image capture. Light from an imaged object passes through the lens and aperture onto the camera sensor.

An illustration of the image acquisition hardware is provided in Figure 4. In this work, a FLIR Blackfly 1” sensor machine vision camera with imaging resolution of 5472 × 3648 pixels were fit with an $F_{l}$ = 8 mm focal length lens. Together, these provided a feature resolution of ≤0.1 mm/pixel at a working distance of *D* = 350 mm, with a Field of View, FoV, of 574 mm × 383 mm.

The depth of field, DoF, defines the distances at which objects remain in focus:

(2) $$DoF=\frac{2\cdot F_{d}^{2}\cdot f_{n}\cdot C}{F_{l}^{2}},$$

where $f_{n}=\frac{f_{l}}{a_{d}}$ is the f-number is the ratio of the focal length, $F_{l}$, to aperture diameter, $a_{d}$. *C* is the circle of confusion: a subjective limit that defines an acceptable level of loss of focus. It is conventionally calculated by dividing the diagonal size of the camera sensor by 1500.

Equation (2) shows that high f-numbers result in a high DoF, but this comes with trade-offs, such as a decrease in image capture speed (as image sensor exposure is reduced). Particularly high f-numbers can cause diffraction effects in images, while low f-numbers can cause blurring at image edges. In this work, we selected $f_{n}$ = 8 and $F_{d}$ = 250 mm. This resulted in no discernible diffraction effects with acceptable levels of edge blurring. This allows objects to be imaged clearly between lens-object distances of 200 mm to 350 mm.

### 3.2. Dataset Acquisition and Preprocessing

**Figure 5:**
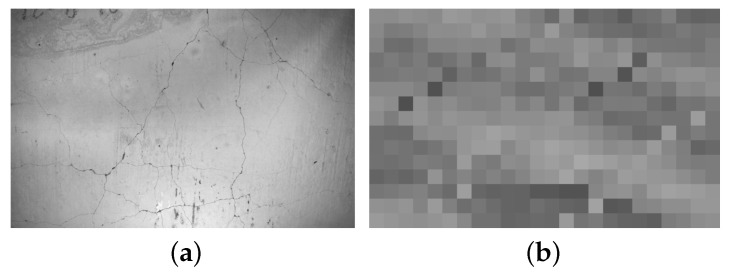
(**a**) Baseline (pristine) image. (**b**) Colour map of pristine image (the lighter the pixels, the better the image quality).

A dataset of high-resolution images of concrete surfaces with visible cracks was obtained for this study. The dataset consisted of damaged, reinforced concrete slabs measuring 500 mm × 500 mm × 10 mm, with crack widths ranging from 0.07 mm to 0.3 mm. The cracks were generated by applying forces at the edges, and the images were captured under diffuse lighting. A preprocessing step was performed to prepare the images for input to the VGG16 model, where the large image shown in Figure 5 was cropped into blocks of size 224 × 224 pixels.

Overall, a data set of 3600 sub-images, each corresponding to a size of 224 × 224 pixels, was curated. Of this dataset, 2100 images displayed cracks, and 1500 images displayed clear concrete surfaces. The images were manually labelled as cracked or not cracked. The “baseline” images were degraded by introducing common degradations such as Gaussian noise and Gaussian blur. These degradations were chosen as they are commonly produced in real datasets during image acquisition and can severely impact crack classification accuracy.

**Figure 6:**
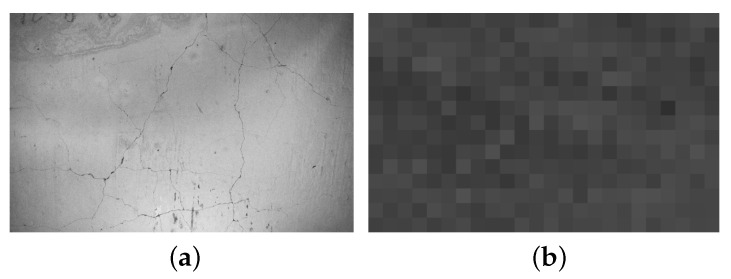
(**a**) Noisy image of noise factor 50. (**b**) Colour map of noisy image (the lighter the pixels, the better the image quality).

In this study, we applied Gaussian noise to the original images by increasing the noise level from 10 to 50; an example of this is shown in Figure 6. In Equation (3), the coordinates (x,y) represent the pixel coordinates, s(x,y) is the original image, n(x,y) is the added Gaussian noise, and w(x,y) is the resulting noisy image [40]. The ‘np.random.normal()’ function is used to generate the Gaussian noise.

(3) w(x,y)=s(x,y)+n(x,y)

**Figure 7:**
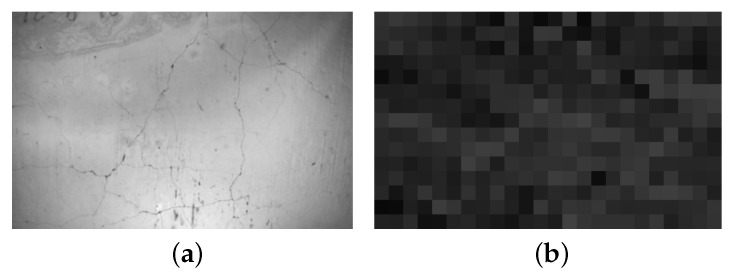
(**a**) Blurred image of blur factor 8. (**b**) Colour map of blurred image (the lighter the pixels, the better the image quality).

In this study, we applied Gaussian blur by varying the blur factor from 1 to 10, with the levels being 1, 2, 5, 8, and 10, as represented by Equation (4), where I is the original image without the blur, $I_{\mathrm{blurred}}$ is the blurred image that contains Gaussian blur, G is a Gaussian kernel with standard deviation σ, and ⊗ denotes the convolution operation [41]. Gaussian blur is achieved by convolving the image with a Gaussian kernel using a function called ‘ImageFilter.GaussianBlur()’. A higher blur factor indicates a higher level of blur applied to the image; an example of this is shown in Figure 7.

(4) $$I_{\mathrm{blurred}}=I⊗G\left(\sigma^{2}\right)$$

### 3.3. VGG16 CNN Model

**Figure 8:**
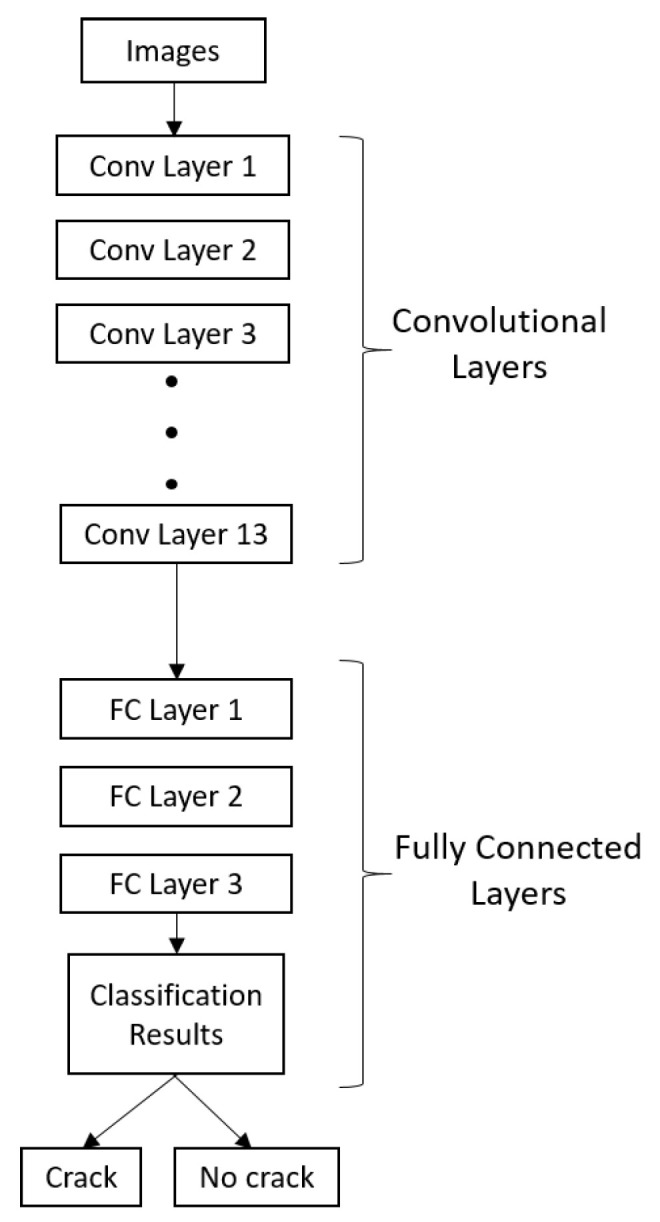
VGG16 crack classification model.

In this study, we utilised the VGG16 CNN model trained on ImageNet as a base model shown in Figure 8. By employing transfer learning, we added layers to the pre-trained model to customise it for our specific task (fine-tuning), enabling the model to learn more efficiently and with fewer training examples [42,43,44,45].

To perform binary classification of concrete images, we utilized transfer learning and loaded a pre-trained VGG16 model that has been previously trained on a large ImageNet dataset. By leveraging the learned features from the VGG16 model, we can save time and resources by avoiding the need for extensive training on our specific dataset. To fine-tune the model, we strategically decided to freeze all the layers except the last six layers of the base model. Our approach, which combined transfer learning, selective fine-tuning, and specialized classification layers, played a pivotal role in achieving state-of-the-art results in our research.

We used the Adam optimiser [46] with binary cross-entropy loss and accuracy metrics to compile the new model. The model was trained for 10 epochs, with a batch size of 32, and then evaluated on the testing data. Dropout was applied in the network architecture to prevent overfitting, and a dense layer with sigmoid activation was employed for binary classification. The performance of the model was assessed by computing the confusion matrix and performance metrics. The VGG16 model implemented in this study is trained and tested on pristine, noisy, and blurred images.

### 3.4. Performance Metrics

We used separate training and testing datasets to evaluate the performance of the VGG16 model on the baseline, noisy, and blurred datasets. The model was trained on the training dataset and was used to generate predictions on the testing dataset. We then converted the predicted probabilities to binary class labels using a threshold of 0.5. The performance of the model was assessed using a confusion matrix generated by comparing the predicted labels to the actual labels of the testing dataset. The confusion matrix provided information about correctly and incorrectly classified instances, represented by true positive (TP), true negative (TN), false positive (FP), and false negative (FN) values. Finally, these values range from 0 to 1, with 1 indicating good and 0 indicating bad, and are utilised to calculate various evaluation metrics as shown in Table 1 [47].

### 3.5. Testing Matrix Analysis

A testing matrix was created to evaluate the performance of the VGG16 model under different conditions. This matrix outlines the different combinations of training and testing scenarios used during the evaluation, as listed in Table 2. The rows indicate the testing conditions, which include noisy, blurred, and pristine images, while the columns represent the training conditions, including noisy, blurred, and pristine images. The “x” marks in the matrix indicate specific training and testing combinations. For example, “Train on Noisy, Test on Noisy” means that the model was trained on noisy images and then tested on noisy images. Similarly, “Train on Blur, Test on Pristine” means that the model was trained on blur images and then tested on pristine images. Thus, the testing matrix provides valuable insights into the crack detection model’s performance under different training and testing combinations.

## 4. Results

The impact of image degradation on image quality can significantly affect a model’s recognition capabilities. Therefore, it is vital to assess image quality using metrics that are sensitive to various types of image degradations. In this section, we evaluate the sensitivity of the BRISQUE image quality metric to different types of degraded images.

### 4.1. The Sensitivity of BRISQUE to Noise and Blur

In this study, we examined the effect of noise and blur on image quality by analysing the BRISQUE scores and utilising colour maps. Colour maps help to visually evaluate the image quality based on colour intensity. Figure 5a represents the baseline (pristine/original) image and its corresponding colour map of BRISQUE scores, which is plotted after data preprocessing, explained in Section 3.2. As shown in Figure 5b, lighter pixels in the colour map indicate lower BRISQUE scores, indicating better image quality. On the other hand, darker pixels represent higher BRISQUE scores, indicating degraded image quality. Different types of degraded images were analysed to determine their effects on BRISQUE.

We investigated the relationship more deeply by introducing various levels of noise and blur to the original images. Figure 6 and Figure 7 display the images with noise level 50 and blur level 8, respectively, along with their corresponding colour maps of BRSIQUE scores. The colour maps clearly show that regions with greater noise or blur have darker pixels, indicating lower image quality and higher BRISQUE scores.

**Figure 9:**
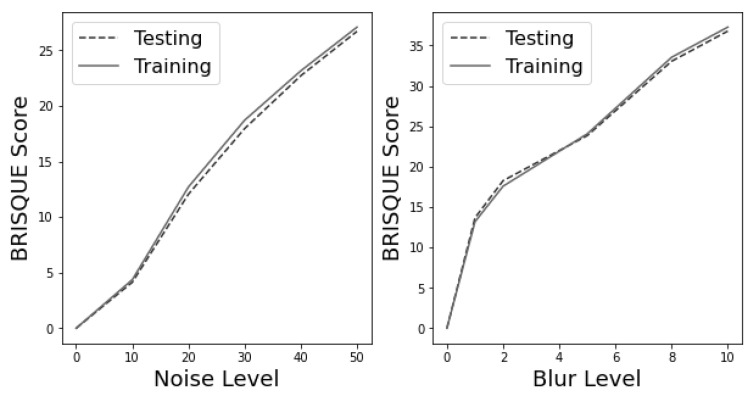
Comparison of BRISQUE scores for various levels of noise and blur in image processing.

Figure 9 displays the BRISQUE scores for different levels of noise and blur. The left subplot illustrates the BRISQUE scores for noise levels ranging from 0 to 50, while the right subplot illustrates the BRISQUE scores for blur levels ranging from 0 to 10. Both subplots feature a normal line representing the training data and a dashed line representing the testing data. As we can see from this plot, the BRISQUE score increases as the noise and blur levels increase, indicating a decline in image quality.

Studying the colour maps and their corresponding BRSIQUE scores provides valuable insights into analysing the impact of noise and blur on image quality. Through these images, it becomes evident that the baseline image has the lowest score. As noise and blur levels increase, the BRSIQUE score rapidly increases, indicating a decline in image quality. Therefore, assessing the quality of the images is imperative, as it significantly impacts the model recognition capabilities discussed in subsequent sections.

### 4.2. Summary of Classification Metrics on Noisy Images

**Figure 10:**
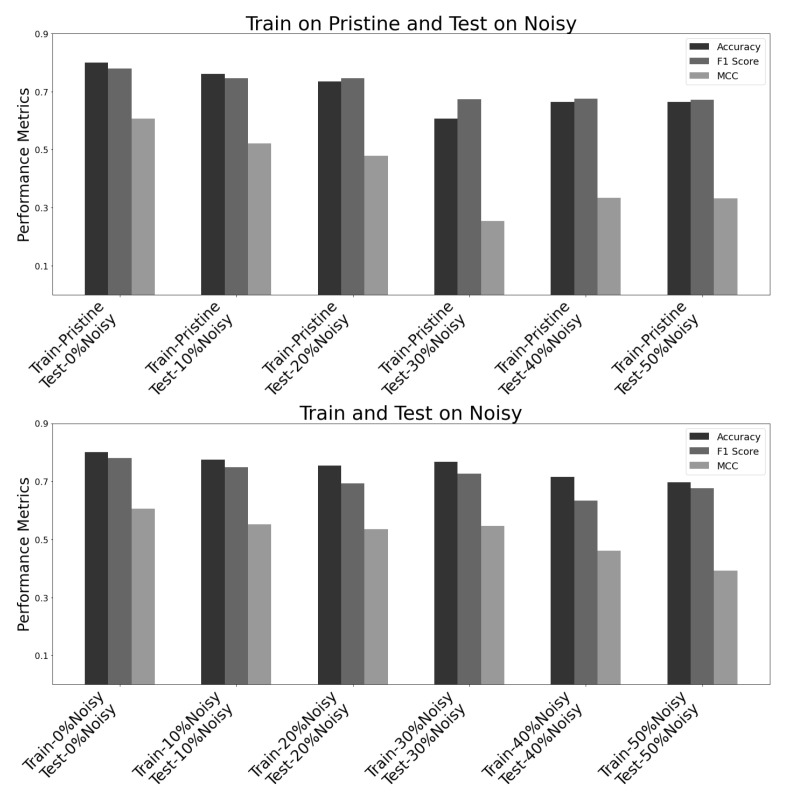
Performance comparison of VGG16 neural network model on Noisy images using accuracy, F1 score, and Matthew’s correlation coefficient metrics.

The VGG16 neural network model was analysed on various datasets using three widely used evaluation metrics, namely accuracy, F1 score, and Matthew’s correlation coefficient. The model underwent training and testing on both original (pristine) datasets and datasets with varying noise levels, as shown in Figure 10 and Table 3. The testing matrix utilised ‘x’ marks to denote different training and testing dataset combinations used to evaluate the model’s performance.

- “Train on Pristine, Test on Pristine” signifies that the model was trained and tested on pristine images, i.e., clear images;
- “Train on Pristine, Test on Noisy” signifies that the model was trained using pristine images and then tested on images with varying noise levels;
- “Train on Noisy, Test on Noisy” signifies that the model was trained and tested on images with varying noise levels.

The graph in Figure 10 shows that the X-axis represents various datasets, as listed in Table 3. These datasets were used to train and test the VGG16 model. The bar graph above, labelled “Train on Pristine and Test on Noisy”, indicates that the model was trained on a clean dataset and then tested on six datasets with increasing noise levels, ranging from 0% to 50%. On the other hand, the bar graph below, labelled “Train and Test on Noisy”, shows that the model was trained and tested on datasets with increasing noise levels, ranging from 0% to 50%. In this case, 0% noisy dataset constitutes pristine (original) and clear images. The Y-axis of the graph represents the performance of each model, as measured by three metrics—accuracy, F1 score, and MCC. The black bars represent accuracy, the dark grey bars represent the F1 score, and the light grey bars represent MCC.

Based on the data presented in the graph, it can be observed that the performance of the VGG16 model declines with an increase in the level of noise in the dataset for all three metrics. Statistical analysis of the data were conducted using a one-way analysis of variance (ANOVA) [48], which yielded a *p*-value of 2.92 × 10${}^{-9}$. This low p-value indicates that the results are statistically significant and that the null hypothesis can be rejected. Thus, there is strong evidence to support the alternate hypothesis, which suggests a significant difference between the compared groups. The model was initially trained and tested on the clean, original dataset, and it achieved the highest scores across all three metrics. However, as the level of noise in the dataset increased, the performance of the VGG16 model dropped significantly, with the models trained on the 40% and 50% noisy datasets performing the worst. Regarding the metrics, both the accuracy and F1 score exhibited similar trends, with performance gradually declining as the noise level increased. On the other hand, the MCC demonstrated a more noticeable performance drop as the noise level increased.

Based on the findings, it appears that the VGG16 model is quite sensitive to noise in the dataset. The model’s performance may significantly decrease when the noise level increases. In addition, looking at Figure 9 and examining the BRISQUE scores of pristine and degraded images with varying noise levels, it becomes clear that even a moderate increase in noise levels can lead to significantly higher BRSIQUE scores, indicating lower image quality. Therefore, training and testing deep learning models on high-quality images (lower BRISQUE scores) is always better to ensure accurate crack detection and classification. By comparing the performance of the model on different datasets, we can gain insights into the suitability of the model for real-world scenarios where image quality cannot be guaranteed.

### 4.3. Summary of Classification Metrics on Blurred Images

**Figure 11:**
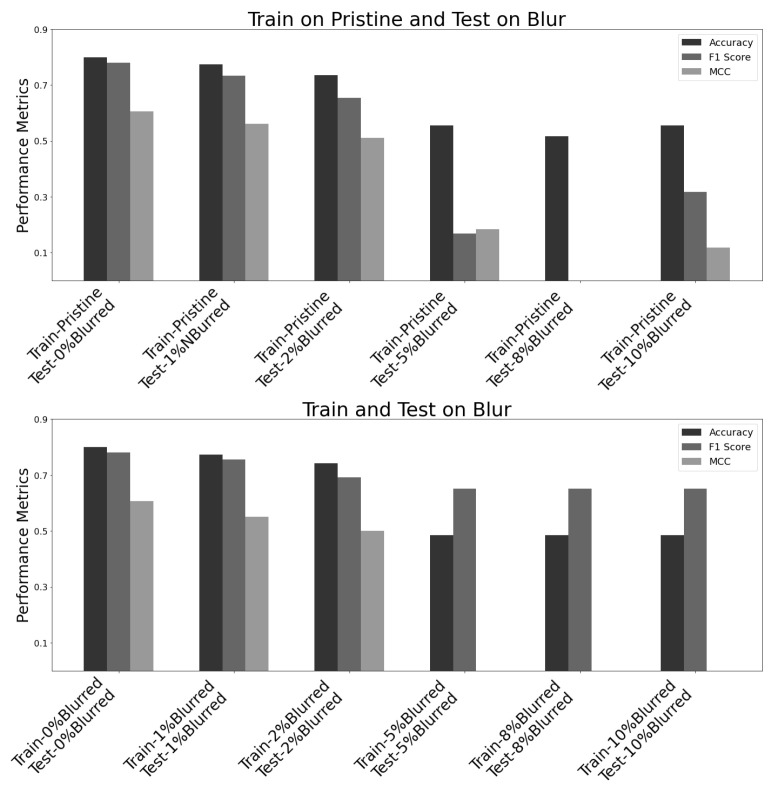
Performance comparison of VGG16 neural network model on Blurred images using accuracy, F1 score, and Matthew’s correlation coefficient metrics.

The VGG16 neural network model was analysed on various datasets using three widely used evaluation metrics, namely accuracy, F1 score, and Matthew’s correlation coefficient. The model underwent training and testing on both original (pristine) datasets and datasets with varying blur levels, as shown in Figure 11 and Table 4. The testing matrix utilised ‘x’ marks to denote different training and testing dataset combinations used to evaluate the model’s performance.

- “Train on Pristine, Test on Pristine” signifies that the model was trained and tested on pristine images, i.e., clear images;
- “Train on Pristine, Test on Blur” signifies that the model was trained using pristine images and then tested on images with varying levels of blur;
- “Train on Blur, Test on Blur” signifies that the model was trained and tested on images with varying levels of blur.

In Figure 11, the X-axis represents the datasets listed in Table 4, which are used for training and testing the VGG16 model. The bar graph above, labelled “Train on Pristine and Test on Blur”, indicates that the model was trained on a pristine dataset but then tested on five distinct datasets with varying levels of blur, ranging from 0% to 10%., and the bar graph below, “Train and Test on Blur” shows that the model was trained and tested on datasets with increasing levels of blur, from 0% to 10%, where 0% blurred dataset represents pristine (original) and clear images. The Y-axis shows the performance of each model, as measured by the three metrics: accuracy, F1 score, and MCC. The black bars represent accuracy, the dark grey bars represent the F1 score, and the light grey bars represent MCC.

Based on the graph, it can be observed that the model’s performance declines in correlation with the increase in the percentage of blur present within the dataset. After conducting a one-way ANOVA on the data, a p-value of 0.0028 was obtained from the statistical analysis. This p-value is considerably low, indicating that the outcome is statistically significant, leading to the rejection of the null hypothesis. This strong evidence supports the alternative hypothesis, which means that the results are statistically significant.

When the model was trained and tested on a pristine dataset without blurring, it achieved the highest scores for all three metrics. However, as the level of blur increased, the model’s accuracy, F1 score, and MCC decreased. For some, F1 and MCC scores were represented as zero in Figure 11, suggesting no true positives were identified in the predictions. This could be due to the model’s inability to correctly classify any images in the testing set, indicating overfitting and poor performance on that dataset.

As shown in Figure 11, the VGG16 model is highly sensitive to blur in the dataset, and its performance may decrease significantly when the blur level is high. This highlights the importance of careful data pre-processing in deep learning applications. Moreover, Figure 9 shows that higher blur levels result in higher BRISQUE scores, indicating lower image quality. This highlights the need to reduce blur in images or remove blurred images before using them in deep learning models. This analysis aimed to assess the suitability of the VGG16 model for real-world scenarios where images may be blurred due to motion or defocus. It underscores the importance of thorough data preparation and cleaning in deep learning applications to ensure high-quality data and optimal model performance.

### 4.4. Optimizing BRISQUE Score Threshold for Crack Detection

This study aimed to determine a BRISQUE score threshold (B${}_{T}$) that could be used to identify and discard images with excessive degradation during preprocessing to improve the performance of the crack detection model. The study utilised a dataset comprising 5665 images. The images were classified into five datasets based on their BRISQUE scores as shown in Table 5. Additionally, each set was divided into train and test sets, and they were manually labelled as either positive (cracks) or negative (no cracks). The VGG16 model was then trained and tested on each dataset, and various metrics were calculated to evaluate its performance.

The performance of the VGG16 neural network model for crack detection and classification was evaluated using different BRISQUE score thresholds ranging from <45 to <85, as shown in Table 5. The analysis revealed that the model’s optimal performance was achieved when the BRISQUE score threshold was set to <45, despite the limited availability of training images. However, the model’s performance gradually decreased as the BRISQUE score threshold increased from <55 to <85. This decrease was not consistently linear due to variations in the number of training images used.

In general, we know that the performance of a model is positively correlated with the number of training images. In particular, increasing the training set size from 720 to 2160 images has improved model performance for BRISQUE score thresholds <55 and <65. However, it should be noted that this positive correlation does not always hold. The performance of the model decreased when the dataset included images with BRISQUE scores <75 and <85. Interestingly, despite having fewer training images, the model achieved its best performance when the BRISQUE score threshold was set to <45. This highlights the importance of image quality in determining neural network performance.

**Figure 12:**
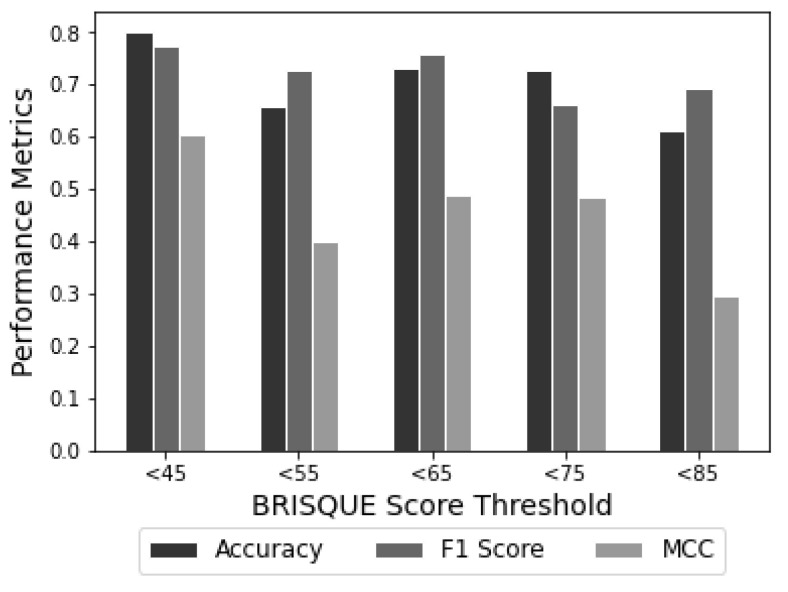
Performance comparison of VGG16 neural network model on images with different BRISQUE score thresholds using accuracy, F1 score, and Matthew’s correlation coefficient metrics.

The distribution of BRISQUE scores is visually represented in Figure 12, which shows distinct score intervals created using histogram binning. The model was trained and tested on images with BRISQUE scores threshold <45 and achieved an accuracy of 0.8, an F1 score of 0.77, and an MCC of 0.606. However, as the BRISQUE score threshold increased, the effectiveness of the model decreased. This implies that the quality of the image greatly influences the accuracy of the crack detection model. It was observed when the threshold of the BRISQUE score was set to <85; the model achieved an accuracy of 0.61, an F1 score of 0.69, and an MCC of 0.30. Hence, images with high levels of degradation can significantly degrade the model’s performance and should be discarded. To summarise, every model has a certain threshold for low-quality images, at which they fail to detect cracks on concrete surfaces. Therefore, allowing only images with BRISQUE scores less than the threshold value helps to improve the crack detection performance. In conclusion, image quality significantly influences the performance of the neural network model for crack detection and classification. The model achieves optimal results when trained on high-quality images, leading to faster training and testing times and lower computational costs by avoiding unnecessary processing of low-quality images.

### 4.5. BRISQUE-Based Data Cleaning

**Figure 13:**
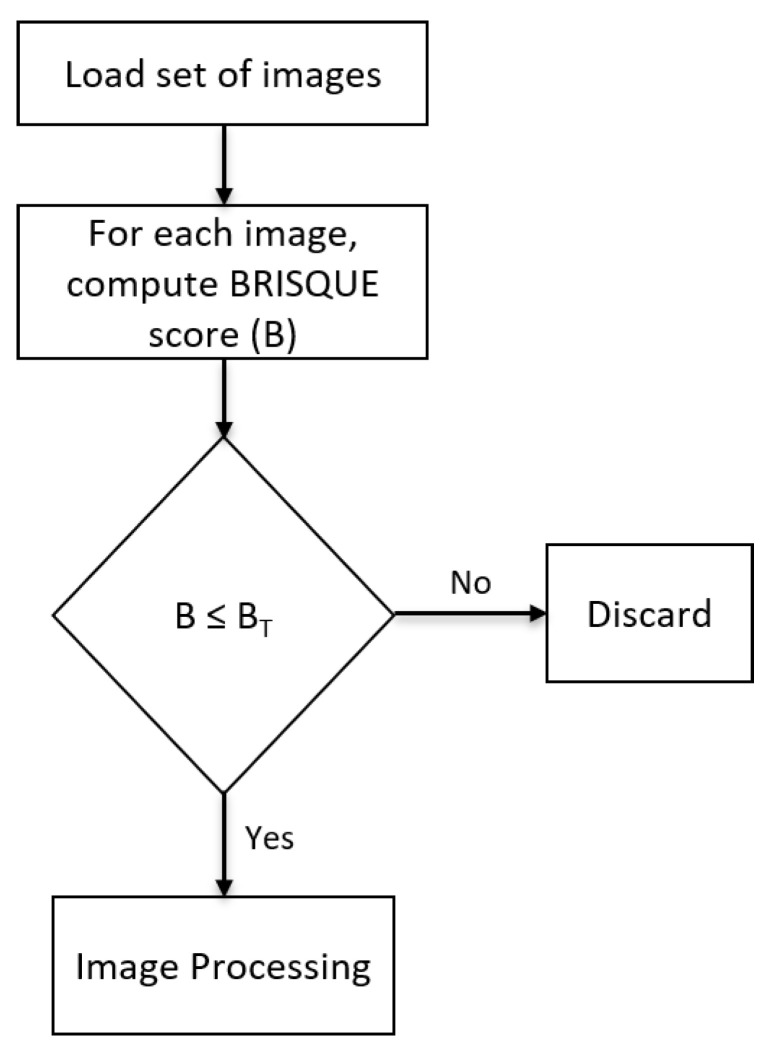
BRISQUE-based data cleaning, where B and B${}_{T}$ represent the BRISQUE score and BRISQUE score threshold, respectively.

To enhance the quality of images utilised for training and testing, it is crucial to carry out data cleaning by removing low-quality images, ultimately saving time. The block diagram presented in Figure 13 illustrates the process of BRISQUE-based data cleaning of images. The algorithm initially loads images, computes the BRISQUE scores (B) for each image, and compares them to a pre-defined threshold value. The BRISQUE score threshold (B${}_{T}$) is determined based on the specific task requirements. If the BRISQUE score of the image surpasses the set threshold, the image is discarded; otherwise, it is retained for further image processing. By eliminating low-quality images, i.e., images with higher BRISQUE scores, the performance and accuracy of the deep learning model can be improved, ensuring that the dataset contains only high-quality images.

#### Impact of BRISQUE Score Threshold (B${}_{T}$) and Data-Cleaning on Model Performance

To maintain fairness and comparability in our model evaluation, we conducted additional testing under uniform conditions, similar to our previous analyses. In this new phase, we acquired 11 images of concrete slabs that were distinct from our prior dataset. These images were captured under uniform conditions and had a resolution of 5472 × 3648 pixels. To facilitate testing, we subdivided each image into blocks of size 224 × 224 pixels.

To ensure consistency, we applied the same BRISQUE score threshold of less than 45, which was successfully used in our earlier experiments. We used a model that had been previously trained on images with BRISQUE scores less than 45, as it demonstrated superior performance in our earlier analyses. We tested this model under two distinct scenarios, one involving data cleaning within a specified BRISQUE score threshold <45 and the other omitting threshold-based data cleaning, thereby including all images, both within and surpassing the BRISQUE score threshold values, i.e., 0<B<100 (includes all BRISQUE scores). This comprehensive analysis provides valuable insights into the model’s adaptability and performance across varying scenarios, ensuring robustness and reliability in our findings.

**Figure 14:**
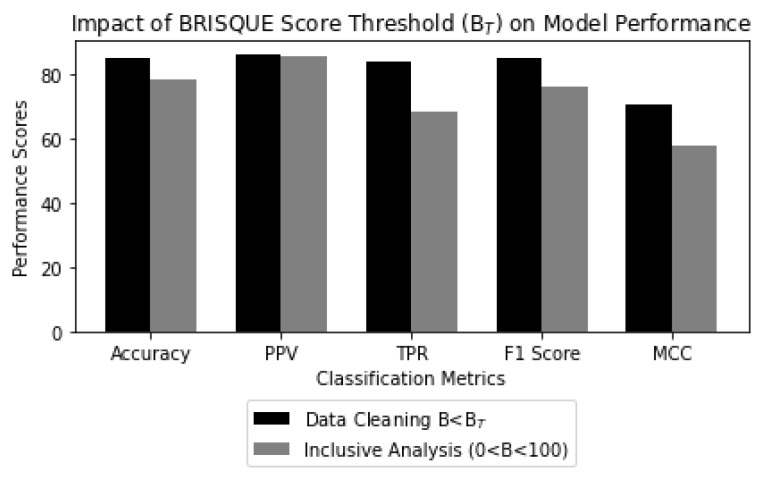
Impact of BRISQUE score threshold (B${}_{T}$) on model performance, where Data Cleaning indicates that the trained model is tested on images within the BRISQUE score threshold, i.e., B < B${}_{T}$, and Inclusive Analysis indicates that the testing dataset includes all images, both within and surpassing the BRISQUE score threshold, i.e., 0 < B < 100.

In the first scenario, we tested the model on the refined dataset containing images with B < B${}_{T}$. In contrast, the second scenario tested the model using a dataset that included all images, regardless of their BRISQUE scores. This inclusive dataset covered a wider spectrum of image quality levels, leading to a noticeable impact on the model’s performance metrics. It is worth noting that the metrics showed significant improvements when data cleaning was conducted within the BRISQUE score threshold, as compared to the scenario without such cleaning as shown in Figure 14. The improvements in classification metrics include:

- A 6.9% increase in accuracy, which indicates that the model classified concrete cracks and non-cracks more effectively, reducing misclassifications;
- A 15.9% enhancement in TPR, which indicates a more effective crack detection rate;
- A 0.6% improvement in PPV, which is relatively modest. However, it signifies a slight reduction in false positives predictions, which further reduces unnecessary inspections;
- A 9.1% improvement in the F1 score, which signifies an overall better performance of the model;
- A 21.5% increase in MCC, which reflects the model’s ability to handle imbalanced datasets.

The significant improvements in metrics observed when data cleaning is performed within the BRISQUE score threshold emphasize the crucial importance of preprocessing in enhancing dataset quality and ultimately leading to superior performance of deep learning models.

## 5. Conclusions and Future Work

It is crucial to assess image quality when detecting and categorising concrete cracks. This study explored the correlation between the BRISQUE-IQA method and the VGG16 crack classification model. The results indicate that the VGG16 model performs best when trained and tested on clean datasets than degraded datasets containing Gaussian noise and blur. The evaluation using BRISQUE scores showed that as the noise or blur in the image increased, the BRISQUE score also increased, indicating a decrease in image quality. Therefore, it is essential to carefully preprocess data and select appropriate metrics and models when developing image processing algorithms.

The study also emphasized the crucial importance of data cleaning within the BRISQUE score threshold and its significant role in enhancing model adaptability and performance in diverse image quality scenarios. These findings open new avenues for developing image-based inspection systems, providing valuable contributions to fields that rely on precise image analysis. The BRISQUE score threshold (B${}_{T}$) effectively evaluated image quality and enhanced the model’s classification accuracy.

Incorporating the implemented methodology into the data preprocessing stage reduces the computation burden of processing low-quality images. It also helps in improving the efficiency of the crack detection and classification process, resulting in faster training and testing times and reduced computational costs. The results of this study have important implications for improving the precision and dependability of automated visual inspection systems used to detect concrete cracks and monitor structural health. Moreover, this knowledge can be applied to diverse fields, including medical imaging and face recognition, where high-quality images are critical for accurate diagnoses. Furthermore, the proposed BRISQUE-assisted deep learning approach can quickly screen vast surface areas of concrete structures such as airport runways and highway pavements.

As part of our future research, we plan to expand our dataset by including a broader range of concrete images that contain various real-world confounding variables (e.g., bio-fouling, efflorescence, corrosion, spalling) and conduct an extensive comparative analysis of various image classification networks, including VGG-16, ResNet, Inception, Xception, and others, for concrete image quality assessment.

## Figures and Tables

**Table 1:** Performance metrics of a binary classifier.

Name	Description	Equation
True Positive Rate (TPR)	The ratio of correctly predicted positive instances to the total number of actual positive instances.	$\frac{TP}{TP+FN}$
Positive Predictive Value (PPV)	The ratio of correctly predicted positive instances to the total number of positive predictions.	$\frac{TP}{TP+FP}$
F1 Score	The weighted average of recall and precision.	$\frac{2\times \mathrm{Precision}\times \mathrm{Recall}}{\mathrm{Precision}+\mathrm{Recall}}$
Accuracy	The measure of the model’s ability to correctly predict the outcomes.	$\frac{TP+TN}{TP+FN+FP+TN}$
Matthew’s Correlation Coefficient (MCC)	The measure of the correlation between predicted and actual labels, accounting for true or false, both positive and negative predictions.	$\frac{TP\times TN-FP\times FN}{\sqrt{\left(TP+FP)\cdot (TP+FN)\cdot (TN+FP)\cdot (TN+FN\right)}}$

**Table 2:** Testing matrix.

	Train on Noisy	Train on Blur	Train on Pristine
**Test on** **Noisy**	x		x
**Test on** **Blur**		x	x
**Test on** **Pristine**			x

**Table 3:** Testing matrix when the model is trained and tested on noisy images.

	Train on Noisy	Train on Pristine
**Test on** **Noisy**	x	x
**Test on** **Pristine**		x

**Table 4:** Testing matrix when the model is trained and tested on blurred images.

	Train on Blur	Train on Pristine
**Test on** **Blur**	x	x
**Test on** **Pristine**		x

**Table 5:** Comparison of model performance at different BRISQUE score threshold values.

BRISQUE Scores	Number of Training Images	Accuracy	F1 Score	MCC
<45	360	0.8	0.77	0.61
<55	720	0.66	0.73	0.40
<65	2160	0.73	0.76	0.49
<75	3240	0.73	0.66	0.48
<85	3960	0.61	0.69	0.30

## Data Availability

The data that has been used in this study is confidential and is unavailable due to privacy or ethical restrictions.
